# Effects of Trigonelline, an Alkaloid Present in Coffee, on Diabetes-Induced Disorders in the Rat Skeletal System

**DOI:** 10.3390/nu8030133

**Published:** 2016-03-02

**Authors:** Joanna Folwarczna, Aleksandra Janas, Maria Pytlik, Urszula Cegieła, Leszek Śliwiński, Zora Krivošíková, Kornélia Štefíková, Martin Gajdoš

**Affiliations:** 1 Department of Pharmacology, School of Pharmacy with the Division of Laboratory Medicine, Medical University of Silesia, 40-055 Katowice, Poland; ajanas@sum.edu.pl (A.J.); mariapytlik@gmail.com (M.P.); ucegiela@o2.pl (U.C.); lsliw@o2.pl (L.Ś.); 2 Department of Clinical and Experimental Pharmacotherapy, Medical Faculty, Slovak Medical University, 833 03 Bratislava, Slovakia; zora.krivosikova@szu.sk (Z.K.); kornelia.stefikova@szu.sk (K.Š.); martin.gajdos@szu.sk (M.G.)

**Keywords:** trigonelline, coffee, streptozotocin, diabetes, skeletal system, rats

## Abstract

Diabetes increases bone fracture risk. Trigonelline, an alkaloid with potential antidiabetic activity, is present in considerable amounts in coffee. The aim of the study was to investigate the effects of trigonelline on experimental diabetes-induced disorders in the rat skeletal system. Effects of trigonelline (50 mg/kg p.o. daily for four weeks) were investigated in three-month-old female Wistar rats, which, two weeks before the start of trigonelline administration, received streptozotocin (60 mg/kg i.p.) or streptozotocin after nicotinamide (230 mg/kg i.p.). Serum bone turnover markers, bone mineralization, and mechanical properties were studied. Streptozotocin induced diabetes, with significant worsening of bone mineralization and bone mechanical properties. Streptozotocin after nicotinamide induced slight glycemia increases in first days of experiment only, however worsening of cancellous bone mechanical properties and decreased vertebral bone mineral density (BMD) were demonstrated. Trigonelline decreased bone mineralization and tended to worsen bone mechanical properties in streptozotocin-induced diabetic rats. In nicotinamide/streptozotocin-treated rats, trigonelline significantly increased BMD and tended to improve cancellous bone strength. Trigonelline differentially affected the skeletal system of rats with streptozotocin-induced metabolic disorders, intensifying the osteoporotic changes in streptozotocin-treated rats and favorably affecting bones in the non-hyperglycemic (nicotinamide/streptozotocin-treated) rats. The results indicate that, in certain conditions, trigonelline may damage bone.

## 1. Introduction

Diabetes mellitus belongs to the most prevalent chronic diseases. There are two main types of diabetes: type 1 (insulin-dependent), with β-cell destruction and insulin deficiency, and type 2, with progressive defect of insulin secretion on the background of insulin resistance [1]. Both type 1 and type 2 diabetes are associated with the development of numerous complications, including disorders of bone metabolism leading to increased occurrence of bone fractures [2,3].

Lifestyle and dietary factors may significantly affect the development of pathological changes in diabetes (in particular type 2 diabetes). These factors are also essential in the prevention of osteoporosis. An example of a component of the diet, which may affect both type 2 diabetes development and the skeletal system, is coffee. Coffee drinking has been shown to exert numerous health-promoting effects, among others it decreases the risk of type 2 diabetes [4,5]. On the other hand, it is taken into consideration as a risk factor for osteoporosis, although results of different studies are inconsistent [6,7,8,9].

Trigonelline belongs to major components of coffee, where it is present in amounts similar or smaller than those of caffeine [10,11,12]. Trigonelline is also present in fenugreek (*Trigonella foenum-graecum* L.) seed, used in traditional medicine [13]. Trigonelline has been reported to exert several pharmacological activities, including antihyperglycemic and antihyperlipidemic [14].

We have recently reported that trigonelline unfavorably affected the skeletal system of estrogen-deficient rats (a model of postmenopausal osteoporosis), intensifying the development of osteoporotic changes [15]. Trigonelline is present in the diet of coffee-drinkers on a daily basis. Our research hypothesis was that trigonelline may affect the skeletal system in diabetic conditions. Taking into account its antihyperglycemic activity, trigonelline may counteract the development of diabetes-induced bone disorders. However, based on our previous observations, there is a possibility of its unfavorable effects on the skeletal system. The aim of the study was to investigate the effects of trigonelline on diabetes-induced disorders in the rat skeletal system. Two experimental models were used: rats treated with a single dose of streptozotocin, which induced experimental type 1 diabetes [16], and rats treated with streptozotocin after nicotinamide [17], which led to a slight increase in blood glucose level only.

## 2. Materials and Methods

### 2.1. Animals and Chemicals

The experiments were performed on three-month-old female Wistar rats purchased from the Center of Experimental Medicine, Medical University of Silesia, Katowice. The rats were fed a standard laboratory diet (Labofeed B, Wytwórnia Pasz “Morawski”, Poland) *ad libitum*. The protocol for the experiments on animals was approved by the Local Ethics Commission, Katowice, Poland (permission number 81/2013).

Drugs used: trigonelline hydrochloride (Fluka Analytical; Sigma-Aldrich Co., St. Louis, MO, USA) at a dose of 63.3 mg/kg p.o. daily (*i.e*., 50 mg of trigonelline/kg p.o. daily) for four weeks, streptozotocin (Cayman Chemical Company, Ann Arbor, MI, USA), nicotinamide (Sigma-Aldrich Co., St. Louis, MO, USA), ketamine—Bioketan (Vetoquinol Biowet Sp. z o.o., Gorzów Wielkopolski, Poland), xylazine—Xylapan (Vetoquinol Biowet Sp. z o.o., Gorzów Wielkopolski, Poland).

The animals were divided into five groups (*n* = 10):

- Control rats,
- Streptozotocin-treated control rats,
- Streptozotocin-treated rats receiving trigonelline (50 mg/kg p.o. daily),
- Nicotinamide/streptozotocin-treated control rats, and
- Nicotinamide/streptozotocin-treated rats receiving trigonelline (50 mg/kg p.o. daily).

Streptozotocin was administered to the rats of groups II-V in a single dose of 60 mg/kg i.p., dissolved in 0.1 M citrate buffer (pH 4.5; 1 mL/kg). Nicotinamide (230 mg/kg i.p.), dissolved in 0.9% NaCl, was injected (in a volume of 2 mL/kg) 15 min before the administration of streptozotocin in the rats of groups IV and V. Control rats (group I) received the citrate buffer in a volume of 1 mL/kg i.p.

Administration of trigonelline started two weeks after streptozotocin and lasted four weeks. Trigonelline was administered once daily by a stomach tube. All control rats received tap water (the vehicle) at the same volume of 2 mL/kg p.o. The four-week period of trigonelline administration was long enough to demonstrate skeletal effects of trigonelline and other compounds of plant origin in rats [15,18,19].

The blood glucose level in non-fasting animals was examined before the start of the experiment and then once weekly, before trigonelline administration, with the use of Accu-Chek Performa glucometer (Roche Diagnostics GmbH, Mannheim, Germany). The blood sample was taken from tail vessels of conscious rats (by cutting the tail tip). When the glucose level exceeded the upper limit of detection (600 mg/100 mL), this value was taken into the calculations. The streptozotocin-treated rats (groups II and III) which did not develop diabetes were excluded from the experiment. Three rats died during the experiment. The final number of rats in experimental groups was: 10, 8, 7, 10, and 9 for groups I, II, III, IV, and V, respectively.

The rats were fasted overnight after the last trigonelline or vehicle administration. The next day, the blood glucose level was measured and the rats were anesthetized with ketamine and xylazine, and then sacrificed by cardiac exsanguination. The left and right femurs, left tibia, and L-4 vertebra were isolated. The left femurs, tibias, and vertebrae were weighed. The left femur, left tibia, and proximal part of the right femur from each rat, wrapped in gauze soaked in 0.9% NaCl solution, were kept below −20 °C until the mechanical tests were performed on thawed bones [20].

### 2.2. Biochemical Studies

In the serum obtained at the end of the experiment (after four weeks of trigonelline or vehicle administration), concentrations of osteocalcin (a bone formation marker) and C-terminal type I collagen fragments released from bone during bone resorption were measured using enzyme immunoassays (Rat-MID Osteocalcin EIA and RatLaps EIA, respectively, Immunodiagnostic Systems Ltd., Boldon, Tyne and Wear, UK). Serum concentrations of total calcium and cholesterol were determined spectrophotometrically, using (Pointe Scientific, Inc., Canton, MI, USA) kits.

### 2.3. Bone Mechanical Properties Studies

Bone mechanical properties were determined using an Instron 3342 500N apparatus (Instron, Norwood, MA, USA). The data were analyzed by Bluehill 2 version 2.14 software (Instron, Norwood, MA, USA).

Mechanical properties of the diaphysis of the left femurs were assayed with a three-point bending test as previously described [18,20]. The load was applied perpendicularly to the long axis of the femur at the mid-length of the bone. The load, displacement and energy for the yield point (0.05% offset), maximum load point and fracture load point were determined based on the load-displacement curves obtained for each bone. Moreover, the intrinsic parameters—stress and Young’s modulus—were examined, assuming that the femoral diaphysis was an elliptical pipe, as previously described [18].

Mechanical properties of the femoral neck were examined in a compression test [18], with the load applied to the femoral head along the long axis of the bone. The load causing the fracture of the femoral neck (maximum load) was determined.

Mechanical properties of the proximal metaphysis of the left tibia were measured in a three-point bending test as previously described [18,21]. To perform the test, the proximal epiphysis was removed. The same parameters as for the femoral diaphysis were determined. To calculate the intrinsic parameters, it was assumed that the tibial metaphysis was a circular beam [18].

### 2.4. Bone Mineralization Studies

The left tibia, femur, and L-4 vertebra were lyophilized for nine days and weighed. The bones were then mineralized (ashed) at 640 °C for 48 h in a muffle furnace. The content of bone mineral, bone organic substances, and bone water in the isolated bones were calculated (as the ratios to the bone mass).

Moreover, bone mineral density (BMD), bone mineral content (BMC), and bone area measurements were performed on the lyophilized tibias deprived of the proximal epiphysis and on the L-4 vertebra, using dual energy X-ray absorptiometry (DXA; Lunar Prodigy Advance with Encore 2011 software version 13.60, GE Medical Systems, Madison, WI, USA). All the samples were measured for three times and the average value was used.

### 2.5. Statistical Analysis

Results are presented as the mean ± SEM. Statistical analysis was performed using Kruskal–Wallis ANOVA followed by Mann-Whitney *U* test (Statistica 10, StatSoft). The results obtained in trigonelline-treated rats (group III and V) were compared with those of the appropriate control rats with streptozotocin-induced changes (groups II and IV, respectively). Moreover, results from all groups were compared with those of the healthy control rats (group I). *p* values < 0.05 were considered significant.

## 3. Results

### 3.1. Skeletal Changes in Streptozotocin-Treated Rats

Administration of streptozotocin induced type 1 diabetes in rats; the non-fasting blood glucose levels two weeks after the streptozotocin administration exceeded 400 mg/100 mL and tended to further increase in the next weeks. The body mass of the diabetic rats was significantly lower than that of the control rats through the whole experiment (Table 1).

The levels of the bone resorption marker (C-terminal type I collagen fragments) were significantly increased six weeks after the streptozotocin administration, and the levels of the bone formation marker, osteocalcin, tended to decrease (Table 1).

**Figure 1:**
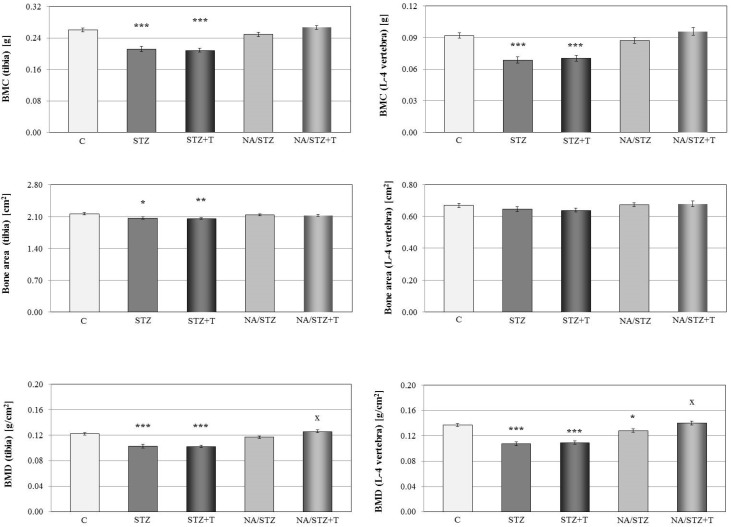
Effect of trigonelline (T; 50 mg/kg p.o. for four weeks) on BMC, bone area and BMD of the tibia (without the proximal epiphysis) and L-4 vertebra in rats treated with streptozotocin (STZ) or nicotinamide and streptozotocin (NA/STZ). Results are presented as means ± SEM (*n* = 7–10). Kruskal–Wallis ANOVA followed by Mann–Whitney *U* test were used for evaluation of the significance of the results. * *p* < 0.05, ** *p* < 0.01, *** *p* < 0.001—significantly different from the control group (C), ^x^ *p* < 0.05—significantly different from the NA/STZ group.

The mass (not shown), BMC and BMD of the long bones and vertebra, as well as the area of the tibia, were decreased in the streptozotocin-induced diabetic rats (Figure 1). The bone mineral mass to bone mass ratio was decreased and the bone water mass to bone mass ratio was increased (with no effect on the mass of organic substances to bone mass ratio) in the femur, tibia, and vertebra (Table 2).

**Figure 2:**
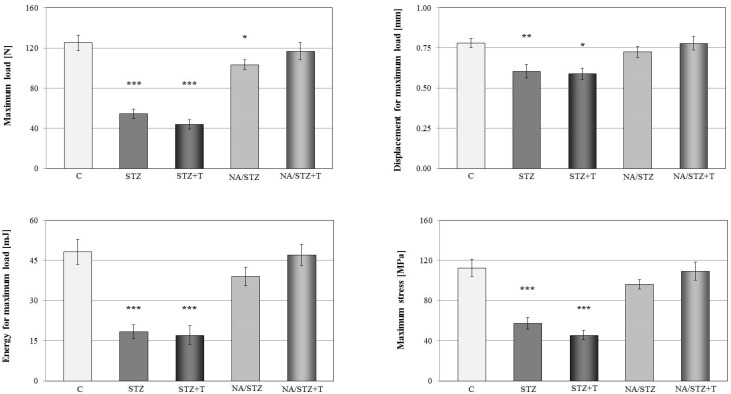
Effect of trigonelline (T; 50 mg/kg p.o. for four weeks) on mechanical properties of the tibial metaphysis (parameters for the maximum load point) in rats treated with streptozotocin (STZ) or nicotinamide and streptozotocin (NA/STZ). Results are presented as means ± SEM (*n* = 7–10). Kruskal–Wallis ANOVA followed by Mann–Whitney *U* test were used for evaluation of the significance of the results. * *p* < 0.05, ** *p* < 0.01, *** *p* < 0.001—significantly different from the control group (C).

Mechanical properties of the tibial metaphysis (mostly cancellous bone) were strongly worsened in rats with streptozotocin-induced diabetes (Figure 2, Table 3). The diabetes-induced changes in compact bone of the femoral diaphysis were much weaker and limited to the parameters measured in the yield point: the yield point load and energy accumulated to the yield point significantly decreased (not shown). There was no effect on the strength of the femoral neck (not shown).

### 3.2. Effect of Trigonelline on the Skeletal System of Rats with Streptozotocin-Induced Diabetes

Administration of trigonelline (50 mg/kg p.o. daily) for four weeks to the rats, in which the diabetes was induced two weeks earlier, did not affect the body mass and the non-fasting glucose levels in relation to the diabetic control rats (Table 1). There was also no effect of trigonelline on the fasting glucose levels (not shown).

Trigonelline intensified the diabetes-induced worsening of the mineralization of the tibia and femur. Although mass (not shown), BMC, bone area, and BMD were not significantly affected, the ratio of the bone mineral mass to bone mass further decreased and the ratio of the bone water mass to bone mass increased in the long bones in relation to the control rats with streptozotocin-induced diabetes (Figure 1, Table 2). Trigonelline tended to intensify the unfavorable changes induced by the diabetes in the bone mechanical properties (Figure 2, Table 3). There was no significant effect of trigonelline on the serum bone turnover markers (Table 1).

### 3.3. Skeletal Changes in Nicotinamide/Streptozotocin-Treated Rats

The injection of nicotinamide (230 mg/kg i.p.) 15 min before the streptozotocin administration counteracted the development of the diabetes. The control nicotinamide/streptozotocin-treated rats had only increased blood glucose levels at the first days of the experiment in relation to the healthy control rats (by 19.7%, 9.4% and 17.4%, after 3, 7, and 14 days, respectively). The body mass gain was unaffected (Table 1).

After six weeks from the nicotinamide and streptozotocin administration, bone mass (not shown), BMC, and the ratio of the bone mineral mass to the bone mass were slightly lower than in the healthy control rats. BMD in the tibia and vertebra decreased (significantly in the vertebra; Figure 1). The ratio of the bone water mass to bone mass significantly increased in the tibia (Table 2).

The nicotinamide/streptozotocin-treated control rats had slightly worsened mechanical properties of the tibial metaphysis (a significant decrease in the maximum load in comparison with the healthy controls; Figure 2, Table 3). There was no effect on the mechanical properties of the femoral diaphysis and neck (not shown).

There was no effect of nicotinamide and streptozotocin administration on the serum bone resorption marker, and the level of the bone formation marker (osteocalcin) was increased in comparison with the healthy controls (Table 1).

### 3.4. Effect of Trigonelline on the Skeletal System of Rats Treated with Nicotinamide and Streptozotocin

Administration of trigonelline (50 mg/kg p.o. daily) for four weeks to the rats, to which nicotinamide and streptozotocin were given two weeks earlier, did not affect the body mass and the glucose levels in rats. There was also no effect of trigonelline on the serum bone turnover markers (Table 1).

Administration of trigonelline to the nicotinamide/streptozotocin-treated rats induced slight increases in the bone mass, BMC and the ratio of bone mineral mass to the bone mass (changes statistically insignificant; Figure 1, Table 2) in relation to the nicotinamide/streptozotocin-treated control rats. However, BMD in the tibia and vertebra significantly increased (Figure 1).

Cancellous bone mechanical properties of the nicotinamide/streptozotocin-treated rats tended to improve after trigonelline administration (the parameters for the maximum load and fracture points of the tibial metaphysis; Figure 2, Table 3). There was no trigonelline effect on the mechanical properties of cortical bone of the femoral diaphysis (not shown).

## 4. Discussion

Pathogenesis of adverse skeletal effects in diabetes is complex. Type 1 diabetes in humans leads to decreased BMD and increases the risk of fractures. In type 2 diabetes, although the reduction in BMD is not observed, the fracture risk is also increased [22,23,24]. In both types of diabetes, hyperglycemia and increased oxidative stress occur [2,25]; the increased oxidative stress intensifies bone resorption and inhibits bone formation [26]. Moreover, the antidiabetic therapy (thiazolidinediones, SGLT2 inhibitors) may contribute to adverse diabetes effects on the skeletal system [27].

One of the most widely used experimental models of type 1 diabetes is diabetes induced by a single administration (i.v. or i.p.) of streptozotocin in rats or mice, which causes destruction of pancreatic β cells and severe hyperglycemia [16]. In the present study, the streptozotocin-treated rats developed diabetes, with hyperglycemia, polydipsia, polyuria, and decreased body mass. Six weeks after the induction of diabetes, very profound changes in the skeletal system were demonstrated, consistent with previous studies [28,29,30]. Bone mass, mineralization (the ratio of bone mineral mass to the bone mass, BMC, and BMD) and mechanical properties of cancellous bone (the proximal tibial metaphysis) dramatically worsened, whereas the strength of compact bone (the femoral diaphysis) was only slightly affected. The decrease in the relative content of the bone mineral was accompanied by an increase in the bone water relative content. Measurements of serum bone turnover marker levels demonstrated that bone resorption was intensified, and bone formation was decreased, as previously described [30,31].

Administration of nicotinamide before the streptozotocin injection to the rats exerts a dose-dependent protective action on β cells, preventing their complete destruction [17]. Some changes corresponding to type 2 diabetes were demonstrated to develop in this model [32,33]. In the present study, nicotinamide in a dose of 230 mg/kg almost completely prevented the streptozotocin action on β cells, since only slight increases in the blood glucose levels were observed in the first two weeks after nicotinamide and streptozotocin administration. Although diabetes did not develop, apparently due to high nicotinamide dose, slight but significant worsening of bone mineralization (vertebral BMD) and cancellous bone mechanical properties developed in the nicotinamide/streptozotocin-treated control rats during the six-week period of the experiment. According to Masiello [32], even those nicotinamide/streptozotocin-treated rats which did not develop hyperglycemia were glucose intolerant. The skeletal changes observed after nicotinamide/streptozotocin administration might have resulted from some disturbances of glucose metabolism. However, since the design of our study did not enable to confirm this supposition, the direct detrimental effect of the single streptozotocin dose on the skeletal system in those rats cannot be excluded.

There is an established link between bone remodeling and energy metabolism, since insulin regulates osteocalcin secretion and activation, and osteocalcin favors insulin secretion by β cells and insulin tissue sensitivity. Insulin resistance in bone, due to decreased osteocalcin activity, leads to decreased tissue sensitivity to insulin [34,35]. However, in the present study, serum osteocalcin levels were only slightly decreased in the diabetic control rats, and they were significantly increased in nicotinamide/streptozotocin-treated rats in relation to the healthy controls.

Trigonelline, administered to the rats at a dose of 50 mg/kg p.o. daily, did not affect non-fasting blood glucose levels during the whole four-week period of administration both in streptozotocin- and nicotinamide/streptozotocin-treated rats. Previously, trigonelline has been reported to exert antihyperglycemic effect in alloxan-induced (type 1) diabetes in rats and in rat and mouse models of type 2 diabetes [14,36,37,38]. It should be pointed out that, in the present study, the nicotinamide/streptozotocin-treated rats were already normoglycemic when the trigonelline administration started. Another reason that trigonelline effect on the glucose levels was not observed in the present study may be related to trigonelline pharmacokinetics. Trigonelline was reported to be well available in rats, with t_1/2_ of 215.9 min [39]. It is possible that at the moment of the measurement, on the next day after the last trigonelline administration, the antihyperglycemic effect was not present any more. Nevertheless, we were interested mainly in the effect of trigonelline on the skeletal system.

Results of the present study confirmed our hypothesis that trigonelline may influence the skeletal system in diabetic rats. Trigonelline differentially affected the skeletal system in the two models used in the present study: it induced unfavorable effects in rats with streptozotocin-induced diabetes and favorable ones in nicotinamide/streptozotocin-treated rats. In rats with type 1 diabetes induced by streptozotocin, trigonelline worsened bone mineralization (further decreasing the ratio of bone mineral mass to bone mass and increasing the ratio of bone water mass to bone mass in the long bones) and tended to worsen cancellous bone mechanical properties (the tibial metaphysis). On the contrary, trigonelline significantly increased BMD and tended to improve cancellous bone strength in the nicotinamide/streptozotocin-treated rats. Taking into account the diversity of molecular targets of trigonelline, the mechanisms involved in those opposite effects may be speculated.

It is possible that differential modulation of oxidative stress parameters by trigonelline is responsible for its differential skeletal effects in the two used rat models. Increased oxidative stress is involved in development both of osteoporosis [26,40] and diabetic complications [25,41,42]. Trigonelline has been reported to attenuate the oxidative stress in different models of type 2 diabetic rats: Goto-Kakizaki rats [43], neonatally streptozotocin-treated rats [44], and high-fat diet streptozotocin-treated rats [37,45]. The favorable trigonelline effects on bones of nicotinamide/streptozotocin rats in the present study might have also resulted from the amelioration of the oxidative stress. On the other hand, trigonelline is an inhibitor of nuclear factor erythroid derived 2-related factor 2 (Nrf2) [46] and is currently used as a tool compound in studies on Nrf2 [47,48]. Activation of Nrf2 pathway is one of the most important defense mechanisms against oxidative stress. In the presence of Nrf2-inducing chemicals or oxidative stimulus, Nrf2 induces transcription of different antioxidant, detoxification, and metabolic control genes [49,50]. In conditions of highly increased oxidative stress parameters, trigonelline, as an Nfr2 inhibitor, may prevent Nfr2 protective effects, which would explain the intensification of the deleterious skeletal effects of streptozotocin-induced type 1 diabetes in the present study.

Another possible mechanism of the unfavorable trigonelline effect on the skeletal system of the diabetic rats is that it may activate PPARγ [45], similarly to thiazolidinediones, antidiabetic drugs with established deleterious effects on bone [27]. However, trigonelline was also reported to downregulate PPARγ regulated pathways in adipocytes [51].

Results of the present study are consistent with our previous observations [15] that trigonelline unfavorably affected the skeletal system of estrogen-deficient rats (bilaterally ovariectomized), intensifying the development of osteoporotic changes in cancellous bone, but did not adversely affect bones of the healthy control rats. The deleterious skeletal effect had been not expected, since trigonelline was reported to exert estrogenic actions in murine MCF7 (breast cancer) cells [52]. Since trigonelline slightly increased the uterus mass in estrogen-deficient rats in our previous experiment, we speculated that trigonelline acted in those rats as an estrogen receptor modulator with an undesired profile of activity: agonistic in the uterus and antagonistic in bone. Based on those results, we proposed that trigonelline may contribute to deleterious effects of high coffee intake on the skeletal system in humans [15]. Results of the present study support this hypothesis.

Although trigonelline is thermolabile and degrades during the roasting [53], it is still present in roasted coffee (2.8 to 9.6 mg/g in Arabica coffee samples [10]; 5.3 mg/g in strongly roasted espresso-type coffee [53]). Trigonelline is readily absorbed after drinking of coffee brew [54]. It should be pointed out that the trigonelline dose (50 mg/kg p.o. daily), used in the present study in rats, corresponds to a dose of 5 mg/kg in man (350 mg/70 kg), taking into account the usual conversion factor of 10, resulting from the faster rat metabolism. Moderate to heavy coffee drinkers can be exposed to similar trigonelline doses, considering that the trigonelline content in roasted coffee may be of similar order to that of caffeine [10,11], and an upper limit for moderate caffeine intake for healthy adult humans is usually thought to be 400 mg (four cups of coffee) daily [6,55].

## 5. Conclusions

Trigonelline differentially affected the skeletal system of rats with streptozotocin-induced bone disorders, intensifying the diabetes-induced osteoporotic changes in streptozotocin-treated rats and favorably affecting bones in non-hyperglycemic (nicotinamide/streptozotocin-treated) rats. The results indicate that trigonelline, an alkaloid present in coffee, may also differentially affect the skeletal system in diabetic patients. There is a possibility of adverse skeletal effects of trigonelline (coffee drinking) in patients with severe hyperglycemia.

## Figures and Tables

**Table 1:** Effect of trigonelline (50 mg/kg p.o. for four weeks) on the general parameters and bone turnover markers in rats treated with streptozotocin (STZ) or nicotinamide and streptozotocin (NA/STZ).

Parameter/Group		Control	STZ	STZ + Trigonelline	NA/STZ	NA/STZ + Trigonelline
Body mass (g)	Initial	193.8 ± 4.4	194.4 ± 5.6	195.6 ± 3.1	193.4 ± 4.2	191.5 ± 3.1
	2 weeks after STZ administration	202.8 ± 5.0	175.4 ± 7.6 *	182.0 ± 4.6 **	202.1 ± 4.1	199.9 ± 3.3
	6 weeks after STZ administration ^#^	217.2 ± 4.6	177.4 ± 5.5 ***	173.2 ± 7.1 **	216.5 ± 4.2	213.8 ± 3.7
Non-fasting glucose level (mg/100 mL)	Initial	117.1 ± 3.8	119.8 ± 3.1	118.4 ± 5.1	119.7 ± 2.5	115.7 ± 4.1
	2 weeks after STZ administration	103.7 ± 3.6	470.7 ± 22.9 ***	528.7 ± 15.8 ***	121.7 ± 7.4	112.3 ± 4.2
	6 weeks after STZ administration ^#^	111.2 ± 3.6	574.8 ± 16.3 ***	576.7 ± 11.7 ***	109.3 ± 3.0	107.2 ± 2.5
Total cholesterol (mg/100 mL)		36.32 ± 2.28	40.80 ± 3.61	33.44 ± 5.75	34.05 ± 2.43	33.52 ± 3.52
Total calcium (mg/100 mL)		9.88 ± 0.09	8.95 ± 0.40	9.57 ± 0.49	10.03 ± 0.17	10.17 ± 0.29
Osteocalcin (ng/mL)		182.4 ± 16.9	129.1 ± 24.3	179.4 ± 5.6	235.7 ± 25.0 *	243.3 ± 14.1 **
C-terminal type I collagen fragments (RatLaps) (ng/mL)		14.51 ± 1.04	59.25 ± 9.36 ***	58.36 ± 5.13 ***	16.47 ± 0.98	15.60 ± 1.14

Results are presented as means ± SEM (*n* = 7–10). Kruskal–Wallis ANOVA followed by Mann–Whitney *U* test were used for evaluation of the significance of the results. ^#^ The measurements made before the last trigonelline or vehicle administration. The biochemical serum parameters were examined six weeks after the STZ or NA/STZ administration. * *p* < 0.05, ** *p* < 0.01, *** *p* < 0.001—significantly different from the control group.

**Table 2:** Effect of trigonelline (50 mg/kg p.o. for four weeks) on the composition of the femur, tibia, and L-4 vertebra in rats treated with streptozotocin (STZ) or nicotinamide and streptozotocin (NA/STZ).

Parameter/Group		Control	STZ	STZ + Trigonelline	NA/STZ	NA/STZ +Trigonelline
Bone mineral mass/bone mass ratio	femur	0.466 ± 0.006	0.440 ± 0.007 **	0.421 ± 0.004 **^,O^	0.458 ± 0.004	0.462 ± 0.007
	tibia	0.466 ± 0.005	0.441 ± 0.006 **	0.419 ± 0.005 ***^,O^	0.451 ± 0.006	0.467 ± 0.004
	L-4 vertebra	0.429 ± 0.012	0.408 ± 0.006 *	0.394 ± 0.006 **	0.424 ± 0.010	0.436 ± 0.006
Mass of bone water/bone mass ratio	femur	0.289 ± 0.006	0.319 ± 0.008 **	0.337 ± 0.006 ***^,O^	0.301 ± 0.005	0.299 ± 0.008
	tibia	0.275 ± 0.005	0.303 ± 0.007 **	0.332 ± 0.005 ***^,O^	0.295 ± 0.008 *	0.280 ± 0.006
	L-4 vertebra	0.316 ± 0.017	0.337 ± 0.008 *	0.352 ± 0.007 **	0.319 ± 0.015	0.307 ± 0.010
Mass of bone organic substances/bo-ne mass ratio	femur	0.244 ± 0.002	0.241 ± 0.002	0.241 ± 0.002	0.240 ± 0.001	0.240 ± 0.002
	tibia	0.258 ± 0.002	0.255 ± 0.004	0.249 ± 0.002	0.254 ± 0.004	0.252 ± 0.002
	L-4 vertebra	0.255 ± 0.006	0.255 ± 0.003	0.254 ± 0.002	0.257 ± 0.005	0.258 ± 0.005

Results are presented as means ± SEM (*n* = 7–10). Kruskal–Wallis ANOVA followed by Mann–Whitney *U* test were used for evaluation of the significance of the results. * *p* < 0.05, ** *p* < 0.01, *** *p* < 0.001—significantly different from the control group, ^O^ *p* < 0.05—significantly different from the STZ group.

**Table 3:** Effect of trigonelline (50 mg/kg p.o. for four weeks) on mechanical properties of the tibial metaphysis (Young’s modulus and the parameters for the yield point and the fracture point) in rats treated with streptozotocin (STZ) or nicotinamide and streptozotocin (NA/STZ).

Parameter/Group	Control	STZ	STZ + Trigonelline	NA/STZ	NA/STZ + Trigonelline
Young’s modulus (MPa)	3299 ± 287	2971 ± 340	3037 ± 341	3366 ± 252	3436 ± 242
Yield point load (N)	70.1 ± 11.4	31.6 ± 4.7 **	21.8 ± 3.1 **	70.0 ± 8.6	62.0 ± 7.7
Displacement for yield point load (mm)	0.426 ± 0.065	0.278 ± 0.031	0.165 ± 0.028 **^,O^	0.463 ± 0.067	0.375 ± 0.059
Energy for yield point load (mJ)	16.62 ± 4.49	4.15 ± 0.91 *	1.98 ± 0.50 **	17.77 ± 4.69	12.36 ± 3.64
Stress for yield point load (MPa)	62.6 ± 9.8	33.1 ± 4.9 *	23.2 ± 3.9 **	64.7 ± 7.4	58.1 ± 8.1
Fracture load (N)	93.5 ± 6.0	41.2 ± 2.9 ***	33.1 ± 4.1 ***	78.0 ± 4.1	90.9 ± 7.2
Displacement for fracture load (mm)	1.092 ± 0.042	0.859 ± 0.049 **	1.121 ± 0.093 ^O^	1.124 ± 0.041	1.021 ± 0.032
Energy for fracture load (mJ)	79.76 ± 4.81	30.90 ± 3.69 ***	37.10 ± 4.66 ***	72.88 ± 3.75	69.71 ± 2.65
Stress for fracture load (MPa)	83.5 ± 5.6	43.2 ± 3.6 ***	33.9 ± 3.6 ***	73.0 ± 4.6	85.3 ± 7.9

Results are presented as means ± SEM (*n* = 7–10). Kruskal–Wallis ANOVA followed by Mann–Whitney *U* test were used for evaluation of the significance of the results. * *p* < 0.05, ** *p* < 0.01, *** *p* < 0.001—significantly different from the control group, ^O^ *p* < 0.05—significantly different from the STZ group.

## References

[1] American Diabetes Association (2015). 2. Classification and diagnosis of diabetes. Diabetes Care.

[2] Yan W., Li X. (2013). Impact of diabetes and its treatments on skeletal diseases. Front. Med..

[3] Starup-Linde J., Vestergaard P. (2015). Management of endocrine disease: Diabetes and osteoporosis: Cause for concern?. Eur. J. Endocrinol..

[4] Beaudoin M.S., Graham T.E. (2011). Methylxanthines and human health: Epidemiological and experimental evidence. Handb. Exp. Pharmacol..

[5] Jiang X., Zhang D., Jiang W. (2014). Coffee and caffeine intake and incidence of type 2 diabetes mellitus: A meta-analysis of prospective studies. Eur. J. Nutr..

[6] Higdon J.V., Frei B. (2006). Coffee and health: A review of recent human research. Crit. Rev. Food Sci. Nutr..

[7] Cano-Marquina A., Tarín J.J., Cano A. (2013). The impact of coffee on health. Maturitas.

[8] Lee D.R., Lee J., Rota M., Lee J., Ahn H.S., Park S.M., Shin D. (2014). Coffee consumption and risk of fractures: A systematic review and dose-response meta-analysis. Bone.

[9] Li S., Dai Z., Wu Q. (2015). Effect of coffee intake on hip fracture: A meta-analysis of prospective cohort studies. Nutr. J..

[10] Perrone D., Donangelo C.M., Farah A. (2008). Fast simultaneous analysis of caffeine, trigonelline, nicotinic acid and sucrose in coffee by liquid chromatography-mass spectrometry. Food Chem..

[11] Do Carmo Carvalho D., Brigagão M.R., dos Santos M.H., de Paula F.B., Giusti-Paiva A., Azevedo L. (2011). Organic and conventional *Coffea arabica* L.: A comparative study of the chemical composition and physiological, biochemical and toxicological effects in Wistar rats. Plant Foods Hum. Nutr..

[12] Rodrigues N.P., Salva T.J.G., Bragagnolo N. (2015). Influence of coffee genotype on bioactive compounds and the *in vitro* capacity to scavenge reactive oxygen and nitrogen species. J. Agric. Food Chem..

[13] Yadav U.C., Baquer N.Z. (2014). Pharmacological effects of *Trigonella foenum-graecum* L. in health and disease. Pharm. Biol..

[14] Zhou J., Chan L., Zhou S. (2012). Trigonelline: A plant alkaloid with therapeutic potential for diabetes and central nervous system disease. Curr. Med. Chem..

[15] Folwarczna J., Zych M., Nowińska B., Pytlik M., Janas A. (2014). Unfavorable effect of trigonelline, an alkaloid present in coffee and fenugreek, on bone mechanical properties in estrogen-deficient rats. Mol. Nutr. Food Res..

[16] Szkudelski T. (2001). The mechanism of alloxan and streptozotocin action in B cells of the rat pancreas. Physiol. Res..

[17] Masiello P., Broca C., Gross R., Roye M., Manteghetti M., Hillaire-Buys D., Novelli M., Ribes G. (1998). Experimental NIDDM: Development of a new model in adult rats administered streptozotocin and nicotinamide. Diabetes.

[18] Folwarczna J., Pytlik M., Zych M., Cegieła U., Kaczmarczyk-Sedlak I., Nowińska B., Śliwiński L. (2013). Favorable effect of moderate dose caffeine on the skeletal system in ovariectomized rats. Mol. Nutr. Food Res..

[19] Folwarczna J., Zych M., Trzeciak H.I. (2010). Effects of curcumin on the skeletal system in rats. Pharmacol. Rep..

[20] Turner C.H., Burr D.B. (1993). Basic biomechanical measurements of bone: A tutorial. Bone.

[21] Stürmer E.K., Seidlová-Wuttke D., Sehmisch S., Rack T., Wille J., Frosch K.H., Wuttke W., Stürmer K.M. (2006). Standardized bending and breaking test for the normal and osteoporotic metaphyseal tibias of the rat: Effect of estradiol, testosterone, and raloxifene. J. Bone Miner. Res..

[22] Khan T.S., Fraser L.A. (2015). Type 1 diabetes and osteoporosis: From molecular pathways to bone phenotype. J. Osteoporos..

[23] Shanbhogue V.V., Mitchell D.M., Rosen C.J., Bouxsein M.L. (2016). Type 2 diabetes and the skeleton: New insights into sweet bones. Lancet Diabetes Endocrinol..

[24] Oei L., Rivadeneira F., Zillikens M.C., Oei E.H. (2015). Diabetes, diabetic complications, and fracture risk. Curr. Osteoporos. Rep..

[25] Maiese K. (2015). New insights for oxidative stress and diabetes mellitus. Oxid. Med. Cell Longev..

[26] Manolagas S.C. (2010). From estrogen-centric to aging and oxidative stress: A revised perspective of the pathogenesis of osteoporosis. Endocr. Rev..

[27] Meier C., Schwartz A.V., Egger A., Lecka-Czernik B. (2016). Effects of diabetes drugs on the skeleton. Bone.

[28] Silva M.J., Brodt M.D., Lynch M.A., McKenzie J.A., Tanouye K.M., Nyman J.S., Wang X. (2009). Type 1 diabetes in young rats leads to progressive trabecular bone loss, cessation of cortical bone growth, and diminished whole bone strength and fatigue life. J. Bone Miner. Res..

[29] Erdal N., Gürgül S., Demirel C., Yildiz A. (2012). The effect of insulin therapy on biomechanical deterioration of bone in streptozotocin (STZ)-induced type 1 diabetes mellitus in rats. Diabetes Res. Clin. Pract..

[30] Coe L.M., Zhang J., McCabe L.R. (2013). Both spontaneous Ins2(+/-) and streptozotocin-induced type I diabetes cause bone loss in young mice. J. Cell. Physiol..

[31] Thrailkill K.M., Clay Bunn R., Nyman J.S., Rettiganti M.R., Cockrell G.E., Wahl E.C., Uppuganti S., Lumpkin C.K., Fowlkes J.L. (2016). SGLT2 inhibitor therapy improves blood glucose but does not prevent diabetic bone disease in diabetic DBA/2J male mice. Bone.

[32] Masiello P. (2006). Animal models of type 2 diabetes with reduced pancreatic beta-cell mass. Int. J. Biochem. Cell Biol..

[33] Szkudelski T., Zywert A., Szkudelska K. (2013). Metabolic disturbances and defects in insulin secretion in rats with streptozotocin-nicotinamide-induced diabetes. Physiol. Res..

[34] Wei J., Ferron M., Clarke C.J., Hannun Y.A., Jiang H., Blaner W.S., Karsenty G. (2014). Bone-specific insulin resistance disrupts whole-body glucose homeostasis via decreased osteocalcin activation. J. Clin. Investig..

[35] Ferron M., Lacombe J. (2014). Regulation of energy metabolism by the skeleton: Osteocalcin and beyond. Arch. Biochem. Biophys..

[36] Hamden K., Bengara A., Amri Z., Elfeki A. (2013). Experimental diabetes treated with trigonelline: Effect on key enzymes related to diabetes and hypertension, β-cell and liver function. Mol. Cell. Biochem..

[37] Zhou J., Zhou S., Zeng S. (2013). Experimental diabetes treated with trigonelline: Effect on β cell and pancreatic oxidative parameters. Fundam. Clin. Pharmacol..

[38] Kamble H.V., Bodhankar S.L. (2014). Cardioprotective effect of concomitant administration of trigonelline and sitagliptin on cardiac biomarkers, lipid levels, electrocardiographic and heamodynamic modulation on cardiomyopathy in diabetic Wistar rats. Biomed. Aging Pathol..

[39] Cheng Z.X., Wu J.J., Liu Z.Q., Lin N. (2013). Development of a hydrophilic interaction chromatography-UPLC assay to determine trigonelline in rat plasma and its application in a pharmacokinetic study. Chin. J. Nat. Med..

[40] Callaway D.A., Jiang J.X. (2015). Reactive oxygen species and oxidative stress in osteoclastogenesis, skeletal aging and bone diseases. J. Bone Miner Metab..

[41] Saito M., Kida Y., Kato S., Marumo K. (2014). Diabetes, collagen, and bone quality. Curr. Osteoporos. Rep..

[42] Kowluru R.A., Mishra M. (2015). Oxidative stress, mitochondrial damage and diabetic retinopathy. Biochim. Biophys. Acta.

[43] Yoshinari O., Takenake A., Igarashi K. (2013). Trigonelline ameliorates oxidative stress in type 2 diabetic Goto-Kakizaki rats. J. Med. Food..

[44] Ghule A.E., Jadhav S.S., Bodhankar S.L. (2012). Trigonelline ameliorates diabetic hypertensive nephropathy by suppression of oxidative stress in kidney and reduction in renal cell apoptosis and fibrosis in streptozotocin induced neonatal diabetic (nSTZ) rats. Int. Immunopharmacol..

[45] Tharaheswari M., Jayachandra Reddy N., Kumar R., Varshney K.C., Kannan M., Sudha Rani S. (2014). Trigonelline and diosgenin attenuate ER stress, oxidative stress-mediated damage in pancreas and enhance adipose tissue PPARγ activity in type 2 diabetic rats. Mol. Cell. Biochem..

[46] Boettler U., Sommerfeld K., Volz N., Pahlke G., Teller N., Somoza V., Lang R., Hofmann T., Marko D. (2011). Coffee constituents as modulators of Nrf2 nuclear translocation and ARE (EpRE)-dependent gene expression. J. Nutr. Biochem..

[47] Abdo S., Shi Y., Otoukesh A., Ghosh A., Lo C.S., Chenier I., Filep J.G., Ingelfinger J.R., Zhang S.L., Chan J.S. (2014). Catalase overexpression prevents nuclear factor erythroid 2-related factor 2 stimulation of renal angiotensinogen gene expression, hypertension, and kidney injury in diabetic mice. Diabetes.

[48] Barroso E., Rodríguez-Rodríguez R., Chacón M.R., Maymó-Masip E., Ferrer L., Salvadó L., Salmerón E., Wabistch M., Palomer X., Vendrell J. (2015). PPARβ/δ ameliorates fructose-induced insulin resistance in adipocytes by preventing Nrf2 activation. Biochim. Biophys. Acta.

[49] Kumar H., Kim I.S., More S.V., Kim B.W., Choi D.K. (2014). Natural product-derived pharmacological modulators of Nrf2/ARE pathway for chronic diseases. Nat. Prod. Rep..

[50] Hybertson B.M., Gao B. (2014). Role of the Nrf2 signaling system in health and disease. Clin. Genet..

[51] Ilavenil S., Arasu M.V., Lee J.C., Kim D.H., Roh S.G., Park H.S., Choi G.J., Mayakrishnan V., Choi K.C. (2014). Trigonelline attenuates the adipocyte differentiation and lipid accumulation in 3T3-L1 cells. Phytomedicine.

[52] Allred K.F., Yackley K.M., Vanamala J., Allred C.D. (2009). Trigonelline is a novel phytoestrogen in coffee beans. J. Nutr..

[53] Lang R., Dieminger N., Beusch A., Lee Y.M., Dunkel A., Suess B., Skurk T., Wahl A., Hauner H., Hofmann T. (2013). Bioappearance and pharmacokinetics of bioactives upon coffee consumption. Anal. Bioanal. Chem..

[54] Lang R., Wahl A., Stark T., Hofmann T. (2011). Urinary N-methylpyridinium and trigonelline as candidate dietary biomarkers of coffee consumption. Mol. Nutr. Food Res..

[55] Nawrot P., Jordan S., Eastwood J., Rotstein J., Hugenholtz A., Feeley M. (2003). Effects of caffeine on human health. Food Addit. Contam..

